# Midwives’ perspectives about using individualized care plans in the provision of immediate postpartum care in Uganda; an exploratory qualitative study

**DOI:** 10.1186/s12912-023-01512-5

**Published:** 2023-09-22

**Authors:** Mariam Namutebi, Gorrette K. Nalwadda, Simon Kasasa, Patience A. Muwanguzi, Dan K. Kaye

**Affiliations:** 1 Department of Nursing, College of Health Sciences, School of Health Sciences, Makerere University, Kampala, Uganda; 2 Department of Epidemiology and Biostatistics, College of Health Sciences, School of Public Health, Makerere University, Kampala, Uganda; 3 Department of Obstetrics and Gynecology, College of Health Sciences, School of Medicine, Makerere University, Kampala, Uganda

**Keywords:** Midwives, Individualized care planning, Postpartum care

## Abstract

**Introduction:**

Individualized care planning has been slowly integrated into practice in Uganda with minimal documentation of how the concept is applied in providing care. This study explored the perceptions of midwives about the use of individualized care plans (ICPs) in the provision of immediate postpartum care.

**Methods:**

An exploratory descriptive qualitative approach was used in this study. We interviewed fifty midwives from 37 health facilities in Uganda's greater Mpigi region. The midwives, who were certificate and diploma holders, were purposively enrolled in the selected facilities. Deductive content analysis was done based on the COM-B model.

**Results:**

Four themes emerged from the data namely; compatibility, motivation, opportunities, and the midwives’ suggested strategies and targets for improved individualized care planning. The midwives were aware of individualized care planning and they utilized it in their provision of immediate postpartum care especially when assessing clients for risks, preparing clients for referral, caring for Human Immunodeficiency virus (HIV) exposed babies and their mothers, and educating/ supporting first-time mothers (automatic motivation). Having a good nurse-patient relationship, privacy, and ample time to care for the clients were noted as motivators for individualized care plan use, while poor documentation of care, high patient load, and perceived patients’ lack of understanding of the complexities of illness in the immediate postpartum period were the barriers (social opportunity) identified by midwives to the use of individualized care planning.

**Conclusion:**

There are still capability, motivation, and opportunity hindrances to the use of individualized care planning. Staff recruitment, training, and harmonization of the documentation forms may improve the use of care plans in the postpartum period.

**Supplementary Information:**

The online version contains supplementary material available at 10.1186/s12912-023-01512-5.

## Background

Individualized care refers to the process of caring for patients that is tailored to meet the specific individual needs of a particular client as assessed by the midwife and agreed upon with the client [1]. Individualized care planning is the process of drawing up a plan for care by taking into account the patient’s actual and potential diagnoses in order to meet their needs in a holistic manner [2]. For individualized care to be safely delivered, the midwife needs to document the appropriate level of care required by the client, evaluate the client’s response to the care, their current social support while reassessing the client for changes in their needs if admitted plus developing a safe discharge plan when appropriate [3]. As such, developing an individualized care plan entails the use of critical thinking, clinical reasoning, clinical judgement and concepts of evidence based practice [4, 5].

From the inception of nursing care planning in the 1950s, the concept has been applied in the process of providing care in most of the world [4]. The concept evolved in midwifery and is referred to as the midwifery care process [6]. This process is preferably initiated in the antenatal period and continued throughout the childbirth continuum [7, 8]. It includes the personalized goals of the care, the treatment needed or anticipated, education needs, care and support for each individual [9, 10]. The goals of the care should be well defined and clear, measurable, achievable and realistic to the health worker, caregivers and patients [10]. Where gaps in the clients’ understanding of their condition are recognized, client/ care taker education must be prioritized to ensure the client and caretaker fully comprehend and participate in the interventions set out to achieve the goals [11]. This ensures professionalism and makes continuity of care possible when the care is provided by a group of health workers which could result in better health outcomes [12]. Through the use of ICPs, the patients feel seen, heard and empowered to participate in their own care which strengthens the health worker-patient relationships [13] and the client participation may improve both the consistency and quality of support provided to them. It also provides evidence of the care provided that can be used in legal proceedings for the benefit of both the health worker and the clients [14].

The Royal College of Midwives (RCM) and the international confederation of midwives (ICM) call for care to be individualized and yet studies done have reported suboptimal care [6, 15–17]. The Guideline development committee for the NICE guidelines for antenatal care planning, quality midwifery care report and a scoping review on the use of post birth care plans reveal a paucity of studies on the care plan implementation and experiences [17, 18]. They thus call for studies to document the concept’s implementation within midwifery [18, 19]. On the other hand, studies done among nurses assessing the implementation of the care planning concept show gaps in the care plan use. A study done among nurses from Greece showed inconsistencies in the reported and actual use of care plans before and after an education intervention [20]. This shows that despite widespread recommendation of individualized care planning, healthcare providers may not systematically follow the required steps. Individualized care planning is a new concept in Africa and a few studies have been done to assess its application in such settings. Most of these studies were quantitative in nature, and showed suboptimal use of care plans by the nurses [21–23]. For instance, a study conducted in Rwanda found that although 73% of nurses used nursing care plans in their practice, only 56% of them did utilize all the steps of the nursing process [24]. Another study done in Kenya also reported similar gaps between theory and practice before the adoption and integration of the nursing process in clinical practice was done [21].

Whereas in many developed countries care plans have been institutionalized and electronic for decades to ease their use in practice [25, 26], in Uganda, patient case files have been paper-based, with no provisions for including midwifery care planning notes until the recent past. A qualitative study done among midwives working at referral hospitals in Uganda noted that it was difficult for midwives in Uganda to provide individualized care to their clients [27]. In this study, factors that hindered provision of care using ICPs included organizational factors (poor work environment and lack of materials/equipment), personal/professional factors (midwives' attitudes, lack of supervision), and policy issues (related to personal benefits such as remuneration, promotion and retirement).

The immediate postpartum period (first 24 h after the delivery of the baby) is critical in childbirth continuum because most maternal and newborn complications/deaths occur either in the intrapartum or in the postpartum period [28]. It is known that 45% and 60% of the maternal and newborn deaths occur within the first 24 h after delivery [29]. Most of these deaths are preventable and could be averted if the mother-baby pairs are assessed and cared for using individualized care plans. However, many women delivering at the health facilities are discharged without a postpartum check [28, 30]. It is not clear what midwives in the rural settings in Uganda know about care planning and how they use care plans in the provision of postpartum care. This study therefore explored the midwives’ perspectives about the use of individualized care plans in the postpartum period so as to inform the possible development of interventions for the use of individualized care plans in postpartum care provision.

### Theoretical framework

**Fig. 1:**
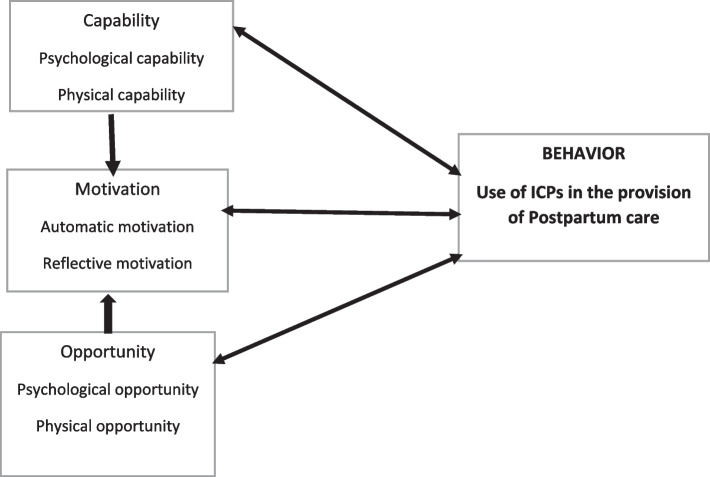
The COM-B Model

This study was informed by the Capability-Motivation-Opportunity for Behavior change model (COM-B) (Fig. 1). The COM-B model conceptualizes behavior as part of a system of interacting factors that explain behavior [31]. According to the COM-B model, for a given behavior to occur, at a given moment, an individual must have the capability and opportunity to engage in the behavior, as well as strength of motivation to engage in the behavior which must be greater than for any other competing behavior [31]. Capability, opportunity, and motivation interact to generate behavior that may, in turn, affect these factors, thereby explaining why a particular behavior is not engaged in, and how behavioral targets can be identified and used as a focus for interventions [31]. In this study, the behavior of interest was the use of individualized care plans (ICPs) in the provision of facility-based postpartum care by midwives. The midwives need to have the physical skills and knowledge about care planning. They also need to have confidence and feel able to provide such care while working in an enabling environment that provides opportunities to provide individualized care. Lastly, the midwives are motivated through rewards or incentives/ punishments related to providing individualized care.

The use of theory offers several advantages [31]: 1) theory summarizes the cumulative knowledge of how to change behavior across different populations, behaviors and contexts. That is, it can inform what interventions may be needed in different contexts, such as for different levels of patient care. 2) Theory-based interventions provide an opportunity in which theory can be tested, thus aiding development of more useful theories which, in turn, supports intervention optimization [32, 33]. That is, theories like the COM-B may inform and enable identification and testing of which interventions may improve individualized care planning. 3) The antecedents of behavior (in this instance individualized care planning), and the causal determinants of / or influences of change can be appropriately identified and targeted by the intervention to address this gap in care. 4) Different strategies can be designed, selected and/or refined and tailored to address specific components of behavior change techniques [33]. These interventions may target health providers, patients, health provider-patient interactions or institutional and health system factors. 5) Theoretically identified mechanisms of action (that is, mediators) can be examined to gain further understanding as to how the individualized care planning intervention brings about its effects [32, 34]. The COM-B model was used to identify the midwives’ perceived capabilities, opportunities and motivation for using individualized care plans in the provision of facility-based postpartum care. This information can be applied to designing interventions to improve the midwives’ use of individualized care plans in their care provision.

## Methods

### Study area

The study was conducted as part of a larger study assessing the provision of postpartum care in the greater Mpigi region in Uganda. The greater Mpigi region in this study consists of the three districts of Butambala, Gomba, and Mpigi which all lie within the central region located between 36-300kms outside of Kampala. The data was collected from selected health facilities that are licensed to conduct deliveries within the region. These included 32 health center (HC) IIIs, three HC IVs and two hospitals.

### Study design

An exploratory descriptive qualitative approach was used to explore the midwives’ perceptions towards the use of individualized care plans in the provision of postpartum care. This study design was chosen because it is useful when a researcher desires to explore the thoughts, the meaning and importance of phenomena that is not well understood [35, 36]. The midwives were from 5 private, 25 public and 7 private not for profit health facilities in the region. The study findings are reported in line with the COREQ guidelines for reporting qualitative studies [37].

### Sampling criteria and participant selection

The study participants were female midwives working in the postpartum units of selected HC IIIs, IVs and, hospitals in the greater Mpigi region. The midwives were selected from 37 health facilities out of the total of 44 within the study area. One to two midwives were interviewed at each HC III, and IV, whereas three to five midwives were interviewed at the two hospitals. This was based on the total number of midwives at each facility and their availability during the time of data collection. All interviews were conducted within the health facilities. On six occasions, midwives requested to be interviewed with a colleague present in the room.

The midwives enrolled in the study were employed in health facilities in Mpigi, Gomba and Butambala districts, had been providing postpartum care for at least 3 months at their current station and consented to participate in the study. At each facility, the midwives were informed about the study and written informed consent sought prior to enrollment in the study. We purposively selected the respondents so as to vary their working experience, level of training and roles played in care provision so as to enrich our data with varying perspectives about individualized care planning from the respondents. These included; maternity ward in-charges, regional trainers and midwives who are dedicated to bedside care. All midwives who were on leave or out of station at the time of the study were excluded from the study.

### Data collection procedure

The in-depth interview guide (Appendix I) used for the face to face interviews was designed by MN following review of literture. It was checked for face and content validity by DKK and GKN. The guide had two sections including the introductions/social demographic characteristics, knowlegde about individualised care planning and perceptions about its importance, application, feasibility and barriers to its implementation. The interview guide was pilot tested with three midwives working at a national referral hospital which was not included in the study. This study is part of MN’s PhD work which is focused on immediate postpartum care hence our interviews did not explore the use of care plans in other dimensions of the childbirth continum.

Data were collected by MN and three research assistants. Two of whom were male graduate nurses and a female who had a diploma in nursing. All the research assistants and MN had prior experience of working in different delivery units providing postpartum care ranging from one year to over ten years. A 3-day research ethics and interviewing skills training was conducted by MN for all the research assistants prior to the commencement of data collection. All interviews were conducted in a quiet place at the facility away from distractions that was identified by MN. All interviews were audio recorded with the participants’ consent and each lasted between 45–90 min. Respondents were enrolled till data saturation was reached (when no new data was being generated from the interviews). Midwives from three health facilities were not interviewed because they declined participation citing heavy workloads on the day the facilities were visited.

### Data management and analysis

The transcribed interviews were read and re-read by DKK, GN and MN. MN checked the transcripts against the audio files and the field notes to ensure consistency of transcription. The transcripts were uploaded into NVivo software [38] by the MN for ease of data management. Deductive content analysis was done based on the eight steps by Yan Zhang and BM Wildemuth. [39]. The data was prepared and analysis done based on the three antecedents of the COM-B model (capability, motivation and opportunities). MN and DKK developed a coding scheme based on the COM-B model and used it to analyze the first three transcripts. Based on the emerging themes and concepts, the rest of the transcripts were coded. Coding consistency was checked in all the transcripts and between the different researchers. Agreement on the meanings of the data and emerging categories and codes by all the authors was ensured through discussions. PAM, DKK, GKN, KS and MN all agreed on the final themes identified. These were further clarified and refined through several discussions online. Portions of the participants’ quotations are shared in the results section. The partial results were shared with midwives from one of the health facilities who validated them.

### Trustworthiness of the study findings

With regard to the trustworthiness of our study findings, the data collection team had some experience in collecting qualitative data prior to the inception of the study and were retrained by MN. All the team members held regular debriefing meetings to discuss the data collected and share their field notes to ensure data credibility. We used triangulation of the data collectors, and the researchers analyzing the data plus researcher agreement on the final themes for confirmability. We also share texts containing the participants’ quotations for dependability of the work. We have endeavored to give details of the context in which the study was done and the steps taken during data collection and analysis to ensure transferability.

## Results

We conducted 50 interviews of midwives from 37 health facilities within the greater Mpigi region. The midwives were all females aged between 22 and 49 years with a mean age of 28.8 years and working experience ranging from 1–24 years. The characteristics of the health facilities where they worked are summarized in Table 1.

**Table 1:** Characteristics of the health facilities where the midwives enrolled in the study worked

Variable	Total (*N* = 37) n (%)	HCIII (*N* = 32) n (%)	HCIV (*N* = 3) n (%)	Hospital (*N* = 2) n (%)
**District**				
Mpigi	18 (48.65)	15 (46.87)	2(66.7)	1(50.0)
Gomba	10(27.02)	9(28.13)	1(33.3)	0(0.0)
Butambala	9(24.32)	8(25.0)	0(0.0)	1(50.0)
**Health facility type**				
Government	25(67.6)	22(68.75)	2(66.7)	1(50.0)
PNFP^a^	7(18.9)	6(18.75)	0(0.0)	1(50.0)
PFP^b^	5(13.5)	4(12.50)	1(33.3)	0(0.0)
**Number of Midwives**				
**1 to 2 midwives**	17(45.95)	17(53.1)	-	-
**3 to 4 midwives**	11(29.73)	11(34.4)	-	-
**5 to 14 midwives**	9(24.32)	4(12.5)	3(100.0)	2(100.0)
**Number of deliveries**				
1 to 20 deliveries	11 (37.5)	10(31.3)	1(33.3)	-
21 to 100 deliveries	22(52.5)	21(65.6)	1(33.3)	-
More than 100 deliveries	4(10.0)	1(3.1)	1(33.3)	2(100.0)
**Number of postnatal beds**				
No beds available	5(13.5)	5(15.6)	-	-
1 to 4 beds	22(59.4)	21(66.5)	1(33.3)	-
5 to 9 beds	7(18.9)	6(18.8)	1(33.3)	-
10 or more beds	3(8.1)	-	1(33.3)	2(100.0)

^a^*PNFP* Private not for profit

^b^*PFP* Private for profit

**Fig. 2:**
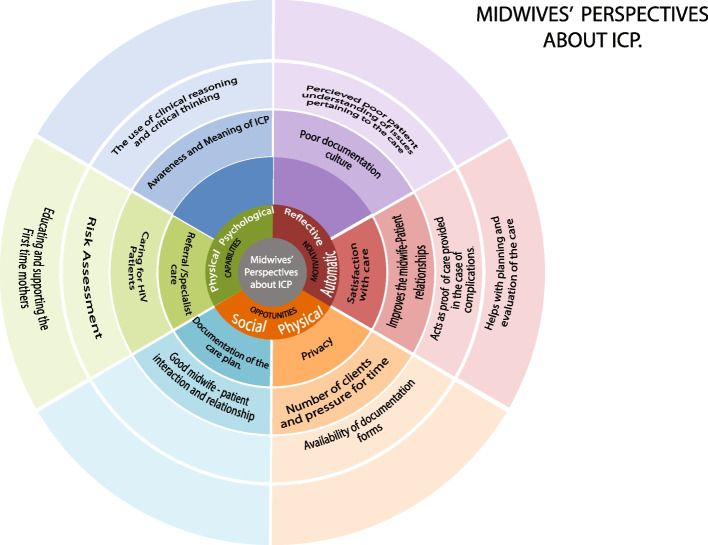
The midwives’ perceptions about ICP, themes and subthemes mapped onto the subcomponents of the COM-B model. This figure has been developed through the modification of the wheel used in the application of the COM-B model to chlamydia testing by LK McDonagh, JM Saunders, J Cassell, T Curtis, H Bastaki, T Hartney and G Rait [40]

There were four themes that emerged from the data analysis namely, Capability, motivation, opportunities for using ICPs and the midwives’ suggested strategies and targets for improved individualized care planning in the immediate postpartum period. These themes and their subthemes are presented in detail in this section. The application of the results to the COM-B model is presented in Fig. 2 and Table 2. Table 3 presents the possible interventions and strategies for improving ICP use.

**Table 2:** Application of the COMB model in our study

Theme	Sub theme	Category	Code
**Capability**	Psychological capability	Awareness and meaning of ICP to the midwives	ICP knowledge
		Use of clinical reasoning and critical thinking	Critical thinking
		Training of midwives	Training
	Physical capabilities	Increasing the number of midwives	Staffing norms
		Assessing the clients’ risk for complications	Risk assessment
		Preparing patients for referral	Referral
		Caring for HIV positive mothers and their newborns	HIV care
		Educating and supporting first time mothers	Health education
**Motivation**	Automatic Motivation	Helps with planning and evaluation of the care	Planning and evaluation
		Improves the midwife patient relationship	Rapport
		Midwives are satisfied with their care	Job satisfaction
		Acts as proof of the care provided in case of complications	Proof of care
		Poor documentation culture	Documentation norms
		Harmonization of the documentation of ICP	Documentation harmonization
	Reflective motivation	Upholding the practice based on the Ideals taught in school	Ideal practice
		Perceived poor patient understanding of issues pertaining to their postpartum care	Perceived patient desires
**Opportunity**	Social opportunity	Documentation of the care	Documentation
		Time and availability of the midwife	Time
	Physical opportunity	Availability of the documentation forms	Available stationery
		Number of clients and pressure for time	Work load
		Good midwife patient interaction and relationship	Midwife-patient interaction
		Privacy	Privacy

**Table 3:** Intervention targets and strategies identified by the participants

Components	Intervention targets	Strategies
**Capability** **Training education and enablement**	Inadequate information about ICP among health workers and clients	**Psychological** • Provide general information about the postpartum period and the possible danger signs for mother and newborn • Provide information about ICP from antenatal clinic • Provide more trainings on ICP with consideration of the health literacy levels of the clients and their care takers
	Inadequate orientation to ICP in both pre-service and in-service trainings	**Physical** • Include ICP in all training curricular • Encourage Midwives to undertake further training • Provide on job training in ICP
**Opportunity** **Environmental restructuring and enablement**	Update all institutional policies to include the use of ICP in care provision	**Physical** • Include ICP forms in the patient documentation files
	Have regular support supervision sessions to support the adoption and use of ICP at all levels	**Social** • Empower supervisors to support midwives in filling and reviewing ICPs
**Motivation** **Incentives, modelling and persuasion**	Ensure all midwives are aware of the benefits of ICP for both the midwife and the clients	**Reflective** • Explain the benefits of ICP to both patients and health workers
	Have champions and role model for ICP at every facility	**Automatic** • Requirement to document care plans for all clients • Provide monetary incentives with ICPs as one of the indicators • Increasing the salaries for midwives

## Theme I: Capability

Under this theme, we present the results in two sub themes namely; the psychological capability and the physical capability of the midwives to provide individualized care using ICPs. These two are described further below;

### Sub theme I: Psychological capability

This has been described as having the knowledge, skills and the psychological stamina to engage in a certain behavior which in our study was providing postpartum care using ICPs. Under this sub theme we had two categories which were awareness and meaning of ICPs to the midwives plus the use of clinical reasoning and creative thinking in the development of ICPs. We elaborate more on these in the paragraphs that follow.

#### Category I: Awareness and meaning of ICP to the midwives

With regards to the meaning of ICP, there was some understanding of what individualized care planning was about among the midwives interviewed. Some recognized that care planning is a cyclic process involving assessment, diagnosis, planning, intervention and evaluation as seen in the quotes below;

*‘What I know about the midwifery decision making. When you see this person, you assess before any diagnosis. You have to assess the person. You have to plan. What you are going to do? You can also make evaluations for the interventions you have implemented; there should be a time frame. What you have done, has it been successful or not? Do we repeat it or not?’* Midwife 4, HC III

*‘You plan, organize and diagnose. I don’t know what is last. Remind us because you are more [telling the interviewer].. So, we are diagnosing, we plan on what to give this mother. Should I admit? What treatment should I give this mother? After that you document, I have given the treatment to such and such a mother; she had malaria.’* Midwife 36, HC III (Public)

Some midwives recognized that adequate planning for postpartum care actually starts in the antenatal period and continues throughout the continuum of maternal-child health care. This is further shown by the quote below;

*‘Midwifery care planning, for me I think, includes care to be given to the mother starting from antenatal clinic when this mother comes onto your clinic; all the services to be given during antenatal care, delivery, care after discharge of that mother.’* Midwife 36, HC III

However not all midwives recalled what individualized care planning was about. In response to an inquiry about the meaning of care planning, several midwives exclaimed that it had been so many years since they had heard about the concept of care planning, and did not know what it entailed, as exemplified by several respondents:

*‘Care plan?.........That was very many years ago!’* Midwife 2, HC III

*‘I am not lying (to) you about that one … I do not have any written.’* Midwife 18, HC III (PNFP)

*‘Care planning? … I don’t know what you really mean.’* Midwife 15, HC III (Public)

“*The problem is that the training on midwifery care plans was a single day and some of us were not on duty”* Midwife 40, Hospital PNFP

#### Category II: Use of clinical reasoning and critical thinking in the development of Individualized care plans

Use of clinical reasoning and analysis was also expressed as a way of applying the findings from patient assessment to the midwifery care process to make decisions for their clients. This was also followed by planning interventions to meet the clients’ needs and eventually evaluation of the outcomes. Below is a quotation from one of the midwives which explain the processes used;

‘*Yes. Still after the delivery, you have to assess your mother. Why? Because you may think that this mother is okay and you leave her around there and you come this way. You see the distance from there up to here is a big one. But all we do is we assess the mother, is the mother okay? ‘Mother, how are you feeling?’ She might tell you that she has a terrible headache. Then there is an action there. Why does this mother have a headache? She might develop. What do we call it? Preeclampsia. There is a way that condition comes about. How does it come about?*’ Midwife 36, HC III (Public)

The midwives in this study noted that care plan development is also guided by the client’s current and potential risks. They highlighted that the patient’s history and current condition can pre-empt their actions and interventions.

*‘For example, they have had a pre eclamptic mother [mother with pre-eclampsia]. This gives you a hint that maybe you need to take her BP [blood pressure], how the mother is doing, bladder emptying/urine output. So, I think it is the way I would prepare the care I would give to my mothers.’ ….* Midwife 38, Hospital (PNFP)

ICP enables providers to plan what care to provide to a given patient or client depending on their clinical presentation. The midwife formulates the care plan and documents it in the patient’s file, as reported by one respondent:

*It depends on the patient’s condition, and what you are managing. Now a woman has come and she has anemia, it’s you who formulates one. Though they give you space for formulating it.* Midwife 37 Hospital (PNFP)

ICP enables planning and preparation for any eventualities, including foresight for likely complications, by analyzing the presentation, causes and likely complications and their management, as the respondents indicated:

*‘You have to be with a plan, in case she gets a complication what do I do, let’s say she completes third stage when she is well and then she enters fourth stage and she gets PPH, what is the care you have to give, you know you have to do resuscitation, if there are clots you remove them, clean her up and give her the necessary medication to get better.’* Midwife 2, HC III (public)

*‘Then you note that this mother might get fits, [eclamptic fits] because she has severe headache. So, you monitor BP. If it is high, you give what you are supposed to give. If you find the pressure very low, you take note of that too. Therefore, after you have ascertained that the BP is not high and neither is it low but the mother still complains of headache, you query. Then you query whether the bleeding was a lot after delivery. That is all assessment. If she bled a lot, then it could have led to anemia because anemia could be a cause of that severe headache. Unfortunately, do we do not check for Hb [hemoglobin level] here.’* Midwife 36, HC III (public)

One midwife felt that there is no need to have written care plans. One should just follow what they know ought to be done rather than taking the time to document the care. This may represent what could be happening with many midwives who work without documented evidence of the care being provided or planned to be provided.

*“…for the care plan…you just use it off head and apply it” Midwife 12 Hospital*

Another midwife noted that part of their communication of the urgency of the client’s condition involved highlighting the patient’s condition on top of her file. This is done to ensure the client is prioritized by all the subsequent health workers providing care on the unit.

*“..It would help her. You can minimize complications. If mother’s file had high risk for sepsis, antibiotics for 5 days.’ They are written in bold letters. Everyone has to take note. You can’t discharge that mother until she has finished the 5 days. Another thing is that you will know that her CBD (continuous bladder drainage) is one week to two weeks. I think it can prevent complications for this mother.”* Midwife 8 hospital (public)

Others communicated the urgency of the specific client verbally as they handed over to the midwives taking over the care from them as one explains;

*“sometimes we make a note on the file … but for most emergencies you fail to get time to record what you did … usually when they hand over to you, you get a verbal report telling you what was missing”* Midwife 12 hospital

Among the key concepts in developing individualized care plans is the fact that the plan is developed in collaboration with the client and any changes therein shared with them. In our study, midwives did not verbalize how they co developed the plans or shared the plans with the clients. Their views on care planning were mostly focused on the professional’s role in developing and implementing the care plan.

*“Individualized care planning means how I organize care for the mother when I am on duty. For example, I may come on duty in the morning, sort the files and know what each mother needs. Various cases need different care. ……. So, I think it is the way I would prepare the care I would give to my mothers.”* Midwife 12 Hospital (Public)

### Sub theme II: Physical capabilities

The Physical capability in the COMB model refers to one having the physical skills, strength and stamina to perform a given behavior (which is providing postpartum care using ICPs in this study). The midwives in this study applied ICP in the provision of care during the postpartum period. They thus use the concept at various times during their practice. They reported that the use of ICPs was common in certain scenarios that are presented in the six categories below;

#### Category I: Assessing the client’s risk for complications

The midwives noted that they are constantly assessing the women and their babies for complications and risks for complications so as to determine the level of care that they needed after delivery. This was especially important at the time (approximately 1–2 h after delivery) when they are making the decision about whether or not to transfer the mother and baby to the postnatal area. The midwives’ quotes below explain the decision making process;

*‘Stable, is this mother who is conscious, aware of their environment, well conversant with the people she is with and is she breathing well. Because when you take the blood pressures, you will know if the mother is bleeding or the mother is not in a good condition. Take her temperature, is she in shock, so all those things. At times a mother can deliver when she cannot sit, so how can you take such a mother to postnatal ward? Even standing, she is feeling dizzy, she can’t go. So, if this mother can sit, stand, move without support or being guided, then we can say she is stable*’ Midwife 38, HC III

*‘Like when they are still here (monitoring area), it is very close but there (postnatal ward) it may take some time (for the midwife to monitor the patients). You may say let me do it every one hour, or four hours. Because most times these mothers do not easily change the condition. Unless if she is very critical, you can check on her every hour. So when the mother leaves this place to the other room, there is nothing big to worry you. There you do the routine way, like they say blood pressures must be taken every four hours and after that you record them in her file.’* Midwife, 40 HC III

Patients who were deemed to be at risk or already having complications were placed closer to where the midwives could see them and keep an eye on them even in the midst of busy units. Often, they were left within the labor ward or placed near the midwives’ station to keep them within view. Below the midwives explain how this is done;

*‘There (points where the very ill patients are kept)… in the position where midwives are passing by every time. We do not put them* [*high risk clients*] *in the far place. Now like here, every time midwives are passing by.’* Midwife 38, Hospital (PNFP)

*‘When you check on the file and find history of PPH, we keep those* [*women*] *in the first stage room for some time. we keep checking for bleeding.’* Midwife 10, Hospital

#### Category II: When preparing patients for referral

The same concept of individualized care planning is applied when patients are being referred from lower health facilities for specialized care. Clients are asked where they would prefer to be referred before the referral note is written so as to take into consideration their financial capabilities and preferences.

*‘All this is included in our clinical notes. How we manage that patient, all those things are written there. what we have done. Even before referral, we have to show that we have given such and such a drug.’* Midwife 36, HC III (Public)

*‘For private, you first ask a person where they will manage, because you can’t send them to Kawempe (National referral hospital) rather Namirembe (private not for profit hospital), when she won’t afford their fees. When she tells you that she can afford Namirembe, you tell her how they work. If she tells you, yet most times I tell her go to Mulago, yet some fear there, that the waiting time is long, what, such stuff, But, you send her to Mulago based on what you know and what’s there. And the quick help she will receive. Because I know that Mulago gives quick help.’* Midwife 17, HC III (private)

#### Category III: Caring for Human Immunodeficiency virus (HIV) positive mother-baby pairs

Midwives were keen on distinguishing the way they individualize their care in the postpartum period. For example, women who are HIV positive and their newborns do need extra care and follow up. This is emphasized all throughout the care and before discharge. Two midwives expound on the matter below;

*‘After they have given birth, they need care after delivery. These mothers we have different cases for example HIV positive mothers they need to be linked to the mother-baby care point, baby has to be bled for 3 Polymerase chain reaction (PCR) tests, have the first PCR at six (6) weeks, the second PCR at nine (9) months and 3*^*rd*^* PCR is at six weeks after cessation of breastfeeding that is at one (1) year for strictly positive mothers and then a rapid test at 18 months but as we do all these the child has to be on medication, Septrin for children 120 and all PCRs we do them as long as the baby is negative*.’ Midwife 1, HC III

*‘You must also make sure you know her HIV status. We make sure that her status is checked and we know it. Then if the baby needs Nevirapine* [*Antiretroviral drugs*] *then we give her. And also, we re-check her vitals again, before we let her go. Then also, we have to get her treatment before she goes. Aah, I think that is it, I can say!’* Midwife13, Hospital (PNFP*)*

#### Category IV: Educating and supporting the first-time mother

When working with first time mothers, midwives noted that there is a lot more health education required to prepare them for their new role as mothers. This was especially true regarding breastfeeding, newborn care and self-care after delivery as expressed by the following midwives’ quotations below;

*‘It is necessary for one who does not know what to do especially for those who have never given birth to a baby before...… usually they complain that the babies regurgitate the milk when they get cold… they do not know why this happens.’* Midwife 3, HC III (Public)

*‘Our duty is when you go to their ward you need to demonstrate to them how a baby should be held when you are breastfeeding, a prime gravida* [*first time*] *mother fails to know how to breast feed a baby… it is the problem of attachment that is disturbing them, they want to know about breast feeding, cord care of the new born, bathing the new born … then also their personal hygiene. They need to know and be reminded that you need to change the pad.’* Midwife 13, Hospital (PNFP)

*‘Of course, sometimes we witness these babies when they are breast feeding because you might ask a mother, is the baby breast feeding? Yet it’s not, especially when they are prime gravidas (first time mothers), those babies tickle their breasts and they fear to breast feed and prefer the bottle and the other feeds than the breast milk, so you have to witness her feeding the baby*.’ Midwife 30, HC IV (public)

## Theme II: Motivation

These are the internal processes that influence the midwives’ decision making with regard to using ICPs in the provision of postpartum care. These were grouped under the two sub themes as per the COM-B model which are; automatic motivation and reflective motivation. The categories under each sub theme are described below.

### Sub theme I: Automatic motivation

As we analyzed the data there were some factors that enhanced the midwives’ desire to use ICPs and thus led to their impulsive use of the ICPs when providing postpartum care. These were grouped into four categories (*‘helps with planning and evaluating of care’, ‘improves the midwife patient relationship’, ‘midwives are satisfied with their care’ and ‘acts as proof of the care provided in case of complications or audits’) w*hich are explained here.

#### Category I: Helps with planning and evaluating the care

Concerning the benefits of the midwifery care process, midwives noted that it helps them plan the patients’ care, monitor the patients and evaluate the outcomes of their interventions. One midwife elaborates on this below;

*‘Midwifery care plan would first of all help me to manage a mother … midwifery care plan … ……helps you to plan the way you are going to manage a mother if she is going to be in your care for example a mother has eclampsia how am I going to manage her from this time up to this time? so its purpose is to help you be ready and also, to remind you … the mother needs to be given extra attention…so it reminds you … when you refer to it … it reminds you that you had planned to do A, B, C, and D for this mother … it shows you what you have to do and what you last did for the patient.*

*So, the plan can show you that according to this there is an improvement on the patient or there is no improvement, so what is the next plan of action … that is how the midwifery care plan helps!’* Midwife 8, Hospital (public)

*‘Now, considering our patients here in antenatal, we ask them which number of child is this, she says this is the tenth, obviously, you know that this one may get PPH, she is multi gravid (has had many babies), and another says PG (prime gravida), even those PGs most times they get PPH, sometimes she may get a tear inside you didn’t see, …you know this is a risk factor and you’re too keen while managing her, when you’re done and she has delivered, you assess the vulva if its ok, did I suture everywhere properly, are the clots all expelled, though at times the uterus may refuse to contract, so we give them oxytocin to help with the uterine contraction.’* Midwife 5, HC III

The practice of care planning is beneficial for the whole health care team and the mother because it fosters easy identification of women with possible complications which enables the team to pay extra attention to them.

*‘First of all, you identify her by clerking. When she is telling you, you will identify something that you should not overlook. For example, if this mother has 2 previous scars. So, the most important thing is that you have to go to her history. Then the other thing is that you have to put it in her chart, because if you do not put it someone can come and misses it out. So we have to put that risk factor on top of her file for easy identification and attention. And management will be more specific to her condition that is highlighted.’* Midwife 37, Hospital (PNFP)

As a result of the monitoring of the mother and newborn, the midwives noted that they had good maternal and newborn outcomes. This was also because they were able to prevent and manage any complications early hence discharged mother-baby pairs rarely returned seeking care for complications in the early postpartum period as one midwife from a HC III reports below.

*‘I think we benefit, because the truth is I have never received a mother who has delivered and got a postpartum problem or ……..she goes home and in two days, she’s back. Even the babies, you can see on our form also has a side for babies, we monitor them the same way, those who normally come back are those with umbilical cord issues….., but it’s hard to get one who comes back sick suffering from fever*.’ Midwife 2, HC III (Public)

#### Category II: Improves the midwife-patient relationships

Midwives noted that individualized care diminishes the barriers to communication between the midwife and her client resulting in the patients freely expressing themselves. This was said to improve overall care as the midwife below explains;

*‘The care plan helps them …I do not know how to call it … it helps to diminish the fear she might have so that she is free to talk to you. Some women fear to talk to us …one who is open with you can easily share anything about her with you.’* Midwife 3, HC III (Public)

*‘By care I mean that attachment between the midwife and the mother…that is why some women choose to go to a certain hospital expecting to find midwife so and so …* [*the reason*] *why they like that midwife is the care and thoughtfulness ‘you’ give her. She values it … but if you are to bark at her to do something … you should have empathy for them. it is not good not to be bothered by their condition…you should show that you care for their wellbeing … that is why patients conclude that such and such a midwife really cared for us, she was there for us or she was friendly towards us.’* Midwife 2, HC III* (Public)*

#### Category III: Midwives are satisfied with their care

Providing individualized care was said to result in midwives being satisfied with their work and it also challenged them to learn more in order to help their clients so they were knowledgeable about the care they provide. One midwife from a public health facility summed up her experience in the quote that follows;

‘*It is the care plan …so when you care for the woman as you are obliged to do … you explain every procedure …manage the pain …you get that satisfaction and the woman is free with you. Explaining to a patient what she did not know puts more confidence in them about you and they are happy about it and you become more knowledgeable. because it helps the patient and you the midwife.’* Midwife 3, HC III (Public)

#### Category IV: Acts as proof of the care provided in case of complications or audits

There were midwives that thought the main reason for documenting and using care plans was to ensure one had evidence of all they had done for each client which would be useful as evidence in case of any investigation arising from the care provided to a client. This was seen as security against litigation and any unforeseen disciplinary action or patient audits.

***‘****It would be beneficial, just in case you have done everything, but it all fails, you have what to show,… it’s like having a partograph, you can monitor this mother, she goes through the first stage properly and the second stage not so well, or she goes through the second stage properly and the third stage not well, …I think that would be helpful in case you get a problem, …you know the issues of midwifery you wake up one morning and your certificate is taken and yet you’re without blame.’* Midwife 2, HC III (Public)

### Sub theme II: Reflective motivation

This covers the reflective processes the midwives engaged in while making the decision to use or not use the ICPs. The two categories under this subtheme were; poor documentation culture and Perceived poor patient understanding of issues pertaining to their care. The next paragraphs elaborate more on these categories;

#### Category I: Poor documentation culture

The midwives noted that they do not often document their care plans because of the other issues in their work environment that make it hard to document the care. It was noted that though they do that work entailed in providing care, the actual documentation is not done. This is explained in the quote below from two midwives at a busy public hospital.

*‘What I am trying to tell you is that we make them but they are not documented. But we do…laughs…it is difficult to concentrate on documenting whatever you have done before you are called upon to attend to an emergency.’* Midwife 9, Hospital (Public)

*‘There is when one fails to document anything and when you check the patient’s file you find the card blank when you want to follow the patient since she was admitted you get it blank though in actual sense, she has been monitored. That is the challenge; we monitor the mothers but fail to document anything.’* Midwife 8, Hospital (Public)

#### Category II: Perceived poor patient understanding of issues pertaining to their care

Although the midwives knew that providing care using a care plan was beneficial to their clients, they noted that, not all of their clients were willing to learn from them or follow their advice. Some young midwives expressed their frustration in the quotes below;

*‘Yes, in some instances it would help although for some cases it would fail. Instances when you try to explain to the patient and she adamantly refuses to understand…she responds, “I have given birth to 5 children without applying that! You mean it will be different on this sixth child?” It’s like you are endeavoring to change what she has been doing to try something new. Sometimes it is difficult to do…you tell her and she says that she will not do it. Some patients do not want to change…it is like for us midwives; when you are told that this medicine was phased out and she replies, “but me I grew up taking this medicine …” so, even if there is change some people do not like to embrace change.’* Midwife 3, HC III (Public)

*‘Sometimes you are dealing with human nature. You may draw up a plan for this person and she comes and tells you ‘I want to go home!’. She is an eclamptic mother and you are telling her that according to how you are, you need to be admitted such that you can have bed rest, we can take your BP, we can monitor how you are taking your medicine. The mother tells you I am not ready to stay, so I have to go back home. Sometimes they are not ready to stay with us. What challenges us is that when she gets worse, she comes back to you. And you feel disappointed yet you have to work on her.’* Midwife 10, Hospital (Public)

## Theme III: Opportunities

These are the external factors that make it possible for the midwives to use ICPs in the provision of postpartum care. The sub themes under this theme were physical opportunity and social opportunity. These two are the subject of the paragraphs that follow;

### Sub theme I: Physical opportunity

Under physical opportunity we did consider the environmental and external factors that would either make the provision of individualized care possible or not. There were three sub categories under this sub theme (number of clients and pressure for time, availability of documentation forms and privacy) as seen below;

#### Category I: Number of clients and pressure for time

In as much as the midwives desired to provide more individualized care to their clients, they acknowledged that increase in the patient volumes could greatly take up the midwife’s time making it hard for her to do so. This was noted especially where there has been a recent surge in the number of deliveries per day as seen in the quotes below.

*‘Because we don’t have many mothers, it is not difficult to sit with your one or two mothers, and you work on them. But some have many mothers, sometimes she has more than five mothers who have delivered and it gives her a hard time, sometimes you have two beds, as soon as you finish one, you go to the other.’* Midwife 2, HC III (Public)

*‘We used to follow the midwifery care plan... you would fully attend to the woman delivering and give her the care she needs... that is why you receive reports *[from patients]* that this hospital has changed.... you would attend to her and explain to her every procedure, help her with the baby especially cord care.... you would give her time.’ *Midwife 8, Hoapital (Public)

The perceived lack of time to provide the care including health education led to some midwives improvising and providing care to groups of women instead of having one on one sessions with each client. This led to a form of task shifting where the patients and their care takers were recruited to monitor the mothers and their babies for any danger signs while they were on the ward. Two midwives explain how this is done below;

*‘No, now listen, for us we do it for the group [demonstrates how], ‘mothers who have delivered, eeeeh, after sometime pass urine’ …So we tell them to get a bucket and urinate and change the pad. It’s not sitting down and saying mama[mother] (one on one), no, sometimes we don’t have that time’* Midwife 30, HC IV

*‘You tell the mother that every woman who delivers has to lose blood however this blood should be minimal … if you are bleeding profusely, ask your attendant or immediate neighbor to alert us*.’ Midwife 13, Hospital (PNFP)

#### Category II: Availability of documentation forms

Additionally, some midwives noted that the practice of documenting the vital signs for the mother and baby after delivery was recently introduced by the district supervisor when she gave them a form where to record their assessment findings. Before that they were not documenting the postpartum care since they had no form on which to document it.

*‘We here in Butambala, we added it ourselves in the Partograph. Sister ‘Z’* [*Assistant district commissioner for maternal and child health*] *put it for us, when we are monitoring a mother, every day we do it but it wasn’t documented before.’ Midwife 1, HC III*

*‘Use it? The new patients’ files we received have a part of midwifery care plan. Yes, the patient’s file was improved … laughs.’* Midwife 13, Hospital (Public)

#### Category III: Privacy

Some midwives noted that provision of care within an environment that ensured privacy did enhance the communication between the midwife and her clients and has improved the practice of individualized care. This was especially true for women who are HIV positive as one midwife elaborates below;

*‘Individualized care plan is where ...I take her (the mother) to a private room to provide privacy. There, she opens up. These people have issues sometimes she is on ART (anti-retroviral therapy) without the knowledge of her husband. That’s it. She will request for attention. When you probe and she needs privacy, you take her to a room where you expect privacy and you talk.’* Midwife 5, HC IV (Private facility)

### Sub theme II: Social opportunity

This relates to the cultural norms and social cues that would affect the provision of postpartum care using ICPs within the hospital settings. Under this subtheme, we had two categories namely; ‘good midwife patient interaction and relationship’ and ‘the documentation of the care plans used in the immediate postpartum period’. These categories are further discussed below.

#### Category I: Good midwife-patient interaction and relationship

Midwives recognized the importance of their interactions with the clients in aiding the provision of individualized care for their clients. The establishment of the midwife- patient relationship was seen as key in the provision of care.

*‘Usually, the care /comfort during and after delivery is delivered through the midwife-patient relationship. I think it is best when she has received love and care of the midwife. It is important that they become friends.’* Midwife 3, HC III (Public)

Another midwife highlighted the need to assess the causes of illness and not just look for biological causes of illness. This necessitates the use of other skills in history taking and patient interviewing. The first impressions the patient has of the midwife at the point of arrival for admission were also noted to be key in the initiation of the midwife-patient interactions.

*‘‘Of course, sometimes, relationship, is not about sitting there and counselling someone, even first impression matters a lot. ‘How you welcome the mother?’ The mother has come and she sat down, then you tell her, ‘mother how can we help you?’ What has brought here?... Do you understand me?’* Midwife 30, HC IV

However, whereas all midwives thought that individualized care was possible, they all agreed that it requires extra time to accomplish. It was acknowledged that the busier the midwife is, the less likely they are to be involved in preparing individualized care plans. Two midwives elaborate on this concept in the quotes below;

*‘Some say it takes time, you need to be available all the time for that management yet if you manage clinically, you put up a drip and do all the rest and leave. The other one (using ICP), you need to be available and put in a lot more time.’* Midwife 7, HC III (PNFP)

*‘Midwifery care plan…(laughs)…to apply it on women? We used to follow it but not of recent. May be when you are not very busy you try to use it … there are days when one is not very busy then you use it.’* Midwife 7, HC III (PPNFP)

#### Category II: Documentation of care plans used in the immediate postpartum period

There was some consensus that documentation of care plans was not a common practice in the facilities. The midwives knew what to do and did it for their clients though it was rarely documented in the patient files or books.

*‘But you see, we might not implement the midwifery diagnosis directly but we apply it.*

*For example, like that case. We examine the mother and diagnose after assessing. You make your plan…We apply it indirectly by going through that process. But when you want to follow the patient since she was admitted you get it (the file) blank though in actual sense, she has been monitored*.’ Midwife 36, HC III (Public)

*‘In each and every case, the midwife has to make some interventions. Yes, we do use the Midwifery care plan although our problem is documenting.’* Midwife 13, Hospital (PNFP)

Though the midwives did not document the care plans in the format as they were taught in school, they were certain that components of the care plans could be found in the patients’ files since they did document the care they provided from assessment, diagnoses and the care/ interventions done for each client. Some however, emphasized that they do write detailed notes for those clients with complications only especially if they are going to refer them.

*‘Those things are written. As you who is admitting this person, you have to plan for that… admit, do this, do this. If it is medication, give this; at this interval. Document it? ... We are supposed to.’* Midwife 12, Hospital (Public)

*‘I think the concept is something that you shall be able to see* [*documented*] *because when I get a patient, we make our clinical notes with our assessment. We make a diagnosis; all this is included in our clinical notes. How we manage that patient, all those things are written there.’* Midwife 36, HC III (Public)

The documentation of the care plans was mainly practiced for those patients who were expected to need the care plan. A patient who was expected to progress without any event or were assessed to be ‘stable’ was considered as not needing a care plan so the care plans were not drawn for them*.*

*‘If we get such a patient (who is very ill), you may find it. But if we don’t, we use our partograph, for our mother is completely normal. We monitor our mother and she is okay. We have some other paper where we do the postnatal care (vital observations documentation tool). We have data that we include there. If we find that our mother is okay, then you will find normal things. If she is not okay, we shall document everything and make a referral. You will find everything.’* Midwife 36, HC III (Public*)*

*‘Though also if the patient is very stable, like the woman has come in the second stage (of labor) and delivers, she is well, in most times we do not start it (the midwifery care plan). We usually put it if the patient comes with complications or those we expect to develop complications. So, you start it, if the patient has any complications.’* Midwife 13, Hospital (PNFP)

## Theme IV: Midwives’ suggested strategies and targets for improved individualized care planning

Under this theme, we present the midwives’ recommendations regarding the changes that could be made to enhance the utilization of individualized care plans in the provision of postpartum care. The four sub themes were; harmonization of the immediate postpartum care documentation, upholding of the practice based on the ideals taught in the training schools, training of the midwives and increasing the number of midwives. These findings are described below and summarized in Table 3.

### Sub theme I: Harmonization of the immediate postpartum care documentation

Midwives in the study noted that either they did not have forms for the documentation of the immediate postpartum care or each facility had their own version of the documentation forms leading to gaps in the documentation and non-uniformity. They thus called for the harmonization of the documents used for documenting care in the immediate postpartum period similar to what was done for the intrapartum period when the ‘Partograph’ was introduced.

*‘Also, these checklists are needed. We have one for the facility which good but it is better when it is uniform across government health facilities. I might make mine which is not similar with the rest you might come to assess and most items are missing. It should be like a ‘Partograph’ such that even if I went to a private facility, I would find a similar one.’* Midwife 4, HC III

### Sub theme II: Upholding practice based on the ideals taught in training school

Some midwives that recalled being taught about individualized care planning in their pre-service training called for all the midwives to return to the practice they were taught before they were recruited into practice saying that it was based on individualized care and should be upheld. A quote from one midwife trainer below elaborates on the matter;

*‘What I think, we health workers we need to make it a habit to go back to the care we have when we are still in school…. when you’re doing cases, (they) follow up a woman /mother until the end, but when they finish school they say to themselves those ended in school, so sometimes in the end you have come to check on the woman and it’s very late, when they have started gasping, they have bled for some time, the bed is full of blood.’* Midwife 1 HC III

There being no hospital policy or law requiring the documentation of the individualized care plans for each client in their files was seen as a loophole that led to lapses in the documentation of the care. As such, some midwives called for the introduction of policies or requirements for clear documentation of the care provided as seen in the quote from a midwife at a HC III below;

*‘Yes, if we have where to document* [*the care plan*] *what we do and it is required by the authorities it will be done’.* Midwife 28, HC III (PNFP)

### Sub theme III: Training of the midwives

Furthermore, the midwives recognized the need to be trained on the concept of individualized care planning so as to aid them in gaining knowledge and applying the concept in their daily practice. They called for in-service training based at their places of work to refresh their knowledge and skills in making individualized care plans. This is further articulated by the quotes from two midwives from HC IIIs;

*‘To help* [*us*]*, we need to be trained more; CMEs. Those who are knowledgeable should train us more.’* Midwife 4, HC III

*‘I think we need orientation. Because usually we start with clinical management. You might research and find that some do not even know midwifery diagnosis. For example, if someone has malaria, I can give an example of malaria. Supervision is mostly done at OPD to reduce drugs given; poly pharmacy such that they know that with this diagnosis, this is what you do but do not give this and that, they have tried a lot to even bring supervisors to check the registers. ‘Who dispensed these drugs?’ they ask,’ This diagnosis, you are not supposed to give these drugs.’ I think it needs strengthening*.’ Midwife 6, HC III

There was also a plea for all midwives not to be comfortable with their current qualifications only but remain open to learning and enroll for new courses so as to keep up with new knowledge and to improve their practice. One participant relates that this will prevent the midwives from using practices that are not up-t o-date unknowingly as seen in the quote below.

*‘Then on-job training. Then also asto use the guidelines to update our knowledge base but to also upgrade academically otherwise you might have outdated information using outdated practices. You need to continually refresh your mind’* Midwife 4 HC III

### Sub theme IV: Increase the number of Midwives

One of the ways the midwives suggested could help improve the implementation of individualized care planning at their facilities was to increase the number of midwives at the facilities. This would aid in reducing their overall workload and free up time to both plan and document their care for their patients. One midwife from a public hospital explains how this would work in the quote that follows.

*‘If we are to get one midwife to be in the nursery, 2 midwives on postnatal and 2 on labor ward you can follow the midwifery care plan. One can concentrate on the mother delivering, then another on women on postnatal … You will not be called when there is a mother who is bleeding on postnatal … No one from nursery will be calling you when a new born has got a complication … Operating theatre attendants will not call you to receive a baby.’* Midwife 10 Hospital (public)

*‘There’s too much work with little manpower because of no money to pay, if there can be funding of someone and we increase on staffing, work will be done easily, but one person is in charge of delivery, antenatal, monitoring patients, there will be gaps in delivery of care to everyone but if the staff are enough, work will be managed properly and patients will be given enough care other than everything being on one person and if the things we need to use while treating can be increased, work will move on easily’* Midwife 47 HC III (PNFP)

## Discussion

In this study, we used the COM-B model to explore the midwives’ capabilities, opportunities and motivations for using individualized care plans. We identified the midwives’ perceived capability, opportunities and motivations for using ICPs in the provision of immediate postpartum care. Capability in our study meant that the midwives had the knowledge, skills and abilities to develop and use individualized care plans. This capability consists of having the mental state, knowledge and skills, and physical strength to make ICPs and apply individualized care planning in their care provision. Opportunities are the external factors that make individualized care planning possible. Motivations are the internal processes that influence the decision making process and they include the reflective processes (like thoughts about the consequences and outcomes of behavior) and the automatic processes like impulses and inhibition [33].

Regarding capability, some of the midwives in the study had the knowledge and training to make individualized care plans for their clients while others were not able to make ICPs because they lacked the knowledge. The recognized opportunities for using ICPs in our study were found in facilities with few clients, more midwives, privacy for the patients, and where the facility required all midwives to document individualized care plans for all postpartum patients. Concerning motivation, the midwives were motivated to make ICPs when they recognized the benefits of using ICPs like better midwife-patient relationships, and better outcomes for their clients. Other motivators for using ICPs were the need for better documentation of care and the use of the documents as proof of care provided in case of complications and as evidence in court in case of litigation. The use of financial incentives have been used in some developed countries to ensure clinicians provide person centered care and in Uganda, result based financing has been used to improve patient care and outcomes [16, 41]. Similarly, in the care practice for school nurses in the United States of America, the avoidance of litigation has been cited as a motivation for clear documentation of care plans [12].

We found that some midwives were generally aware about the concept of individualized care planning, and applied it in the process of providing care to their clients. Their care was guided by the sequential steps in the midwifery care planning process but the documentation of the care was relegated to only those patients who were first-time mothers, very ill, needed referral or were HIV positive. This may be due to the prioritization and integration of care for HIV positive clients in all the maternal newborn, child and adolescent health programs by the ministry of health and the regular training and supervision done at all levels [42]. Also, since some of the midwives interviewed were from HC III and IV which only provide basic emergency obstetric care, the midwives needed to document their care as part of the referral notes written for all critically ill patients that needed to be referred for specialist care [43]. This could also reflect the midwives’ need to be professional when the care they provided will be vetted by others. This view is collaborated by findings of a study done in Uganda where nurses were aware of the nursing theory constructs and believed that if they documented care as required, they would be recognized as professionals [22]. On the other hand the lack of documentation of care for the rest of the mothers who are considered ‘normal’ could be detrimental because it could result in delays in referral and increased maternal morbidity/ mortality when complications arise [44].

In our study, midwives were found to use critical thinking and clinical reasoning in the assessment, diagnosis and provision of care, despite not developing an ICP. These skills have been said to be a key part of how midwives and other health workers process information and make decisions about the care they provide and does promote the use of past experience, clinical knowledge and synthesis of evidence in practice [3, 5, 7, 45, 46].This is important because providing midwifery care is guided by a deliberate, rational and systematic process that is aimed at meeting the patient’s needs [6, 47]. These processes can only be traced when care is clearly documented, which the midwives in our study noted as a challenge in their work. Keenan et al. [39] also noted, in their book on documentation and the nursing care process, that it was hard to see individualized care planning when patient documentation does not bring it in focus, and they advocate for the use of documentation systems that integrate the care planning in the general documentation of patient care.

Midwives at some facilities reported a recent alteration in the patient files to capture the record of the individualized care plans. This was said to improve their planning and evaluation of the care and also resulted in reported gains in their nurse-patient relationships plus job satisfaction for the midwives. This is an opportunity for increased use of ICPs that needs to be adopted by all the health facilities in the region because the midwives were optimistic that it could lead to better documentation of the postpartum care provided. This has also been found to be true in high income countries where good documentation of patient care was found to reflect better care [48, 49]. However, an observational study on documentation of care showed that midwives and nurses tend to find documentation of care burdensome (and disincentive for use of ICPs) because it takes time away from patient care, which was also true for some of the midwives interviewed in this study [50].

None of the midwives in this study, described the process of co-developing care plans with the clients. This may be due to the lack of awareness of the concept or the fact that they are only aware of care plans that are developed by the care worker and only communicated to the client [51]. Another reason for this could that the concept was being discussed in relation to in-patient care and possible emergency situations which required more of the professional input, compared to scenarios of caring for patients with chronic conditions over a prolonged period (where this concept of co-developing care plans with clients was developed) [51, 52]. Midwives felt capable of applying individualized care planning for patients with complications during pregnancy or the postpartum period. This applied to those requiring long term care or referral meaning that they found it easier to plan for clients who were likely to remain in care for longer periods compared to those who were expected to be discharged soon. This is in line with the use of care plans in other countries where individualized care planning is often emphasized for patients that need care for chronic illness or follow up for behavior change programs [51, 53].

In this study, the perceived benefits of using individualized care plans like the good midwife–patient interactions, increased midwives’ satisfaction with their care and good maternal and newborn outcomes influenced the midwives’ use of the care plans. (These factors reflect enhanced opportunity and motivation to draw up and use ICPs for their patients). Studies done in high income countries have found that individualized care planning enhances the midwife-patient relationship and improves the overall quality of care [48, 54]. The facilitators of ICP in our study were closely linked to the midwives’ perceived benefits of individualized care planning. This may be indicative of a synergistic effect between the concept of individualized care and the midwife-patient relationship especially when the patients were few. The effect of individualized care on the patient experience and quality of care has been documented but its effect on the midwife is little known [17, 55]. An Australian study found that midwives in continuity of care settings (where ICPs are used by the same group of midwives for a long time) were more likely to be satisfied with their work and suffer less burn out compared with counterparts in caseload settings [56].

Regarding barriers to ICP noted in this study, midwives reported that the poor documentation culture in their practice hindered the use of individualized care plans. Poor documentation in midwifery has been reported in other countries as well [22, 49, 57]. Not having well documented individualized care plans could hinder the continuity of care when shift changes occur and nothing is documented about the patient care given by the previous care team. This could dampen the morale of the midwives who may need to use the patients’ records but find none. It could also result in midwifery interventions being left undone, putting the patients at an increased risk for infections, complications, poor experience of care and readmission [58].

Another demotivation for the provision of individualized care in this study was the high patient volumes which have been documented in other studies looking at the midwives’ working conditions in Uganda and the implementation of individualized care in other countries [22, 27, 59, 60]. The poor documentation culture and poor working conditions (automatic motivation) affect the quality of care for the patients because they hinder continuity of care in the postpartum period which has been described as most crucial for the survival of mothers and babies [29, 57, 61]. This may also result in medication errors and some clients not receiving the care as per the guidelines as documented in studies that made an inquiry into midwifery documentation issues[48, 54]. Though some of the respondents in this study called for stricter measures to enforce documentation, A Prideaux [54] argues that, that may not be the answer. He calls for a discourse around the nature of the documentation required. Other midwives in this study called for a harmonization of the patient information files saying that would encourage better documentation. However, looking at the documentation debate from the high income countries and the global deficit in midwives, if this is done without increasing the number of midwives per unit, it may only increase the number of ticked forms filled retrospectively but may not necessarily reflect the quality of the care being provided [15, 22, 62]. As we think about moving towards a documentation system that integrates the individualized care plan, we must engage in dialogues to integrate the midwives’ views about the ideal number of midwives per facility, nature of the documentation required and how it would be structured in the Ugandan context [22, 63].

Midwives in this study noted that divergences between the patients’ and midwives’ understanding of the complexities of illness within the postpartum period (reflective motivation) often hindered their provision of care based on the drawn care plans. These differences may have existed because patients probably did not recognize the seriousness of the possible and present risks for complications within the postpartum period and thus preferred to be discharged or discharged themselves before the midwives were certain that this could be done safely. This study was done in a rural setting where the level of illiteracy among women is high and their knowledge regarding postpartum danger signs could be low [64–67]. The knowledge of postnatal danger signs has been found to be low among postpartum women in several studies and associated with the level of maternal literacy and antenatal attendance [67–69]. This highlights the need for health education regarding the importance of maternal and newborn observations in the immediate postpartum period and the postpartum danger signs for women and newborns starting in the antenatal period.

The midwives also recognized the need for both in-service training and advancement of their education (psychological capability) as ways of enhancing their understanding and the use of individualized care plans in practice. This is may be due to the theory practice gap (that was acknowledged by some participants in this study) which results from the discrepancies between what is taught during midwifery training and the way midwifery is practiced in the rural health facilities. This could result in deficiencies in some skills that are not utilized often like making individualized care plans. This theory practice gap has been lauded by some as necessary for the advancement of midwifery and nursing as professions because it maintains the relevance of midwifery education. On the contrary, other authors argue that when practice lags too far behind the theory it can hinder the development of clinical reasoning and practice for both the newly registered midwives and students especially where the practice is more task based like in the Ugandan context [27, 70]. This highlights the need to narrow the theory practice gap because such skills as clinical reasoning and critical thinking are key in grooming good midwifery practitioners. As such, having implementation tools that aid midwives in translation of evidence into practice is important. And from our study, the call for the harmonization of the patient documentation forms may be the tool required to hasten the translation of individualized care planning from theory into practice [71].

## Implications for policy, research, education and practice

To maximize the potential efficacy of interventions (such as for institutionalizing individualized care planning), it is necessary to understand behavior and behavior change. It is critical to have a theoretical understanding of behavior change, whereby theory represents the assumptions about what human behavior is, the influences on behavior, and the accumulated knowledge of the mechanisms of action (mediators) and influences (the moderators) of change [31]. Theories selected to inform intervention design in this context must provide a systematic means of understanding behavior and be capable of capturing the full range of potential levers of change. Many popular theories of behavior focus on the intra-individual and interpersonal factors of behavior, failing to account for the contribution of complex social and physical environments in which behavior occurs [31].

The study identifies several strategies that are in line with ideal interventions for behavior change [72]. There is a need for emphasis on the concepts of individualized care planning for midwives, for instance, during pre-service and in-service training so as to enhance their capability to utilize the concept in care provision. Dialogues with midwives need to be had by the administrators and policy makers to consider ways of enhancing documentation of care, harmonization of the patient information charts and the policies for the implementation of individualized care planning in the rural health facilities. This is important in improving the quality of care across the childbirth continuum and could enhance the patients’ experience of care. Midwives in this study acknowledged that ICP improves their relationships with their clients and enhances their satisfaction. As such, efforts should be made to ensure ICP is practiced in all the health facilities to improve the midwives’ level of satisfaction with their work. This could also be a way of bridging the theory practice gap that was highlighted by midwives in our study. Research needs to be done to explore ways of adapting individualized care planning so that it can be easily utilized by midwives in low resource settings. In Table 3, we highlight the possible intervention targets and the strategies that could be used in research and practice to enhance the use of ICPs in the provision of individualized facility based postpartum care.

## Strengths and limitations

This is one of the few studies done in Africa that adds to the knowledge about the midwives’ perceptions about using individualized care plans during the immediate postpartum period. There was triangulation of data collectors and researchers involved in the analysis of the data for data credibility. We also conducted a large number of interviews with midwives from varied work settings; HC III, IV and hospitals. We also use the participants’ quotations in the reporting of the results. However, we were unable to conduct focus group discussions with the midwives which could have enriched our data because of difficulties in finding a suitable time to gather the midwives without interrupting the provision of care. Also, the study was conducted in one region of the country hence the findings may not reflect the perspectives of midwives in other parts of the country. The perceptions presented in this study are from midwives with either a certificate and/or diploma in midwifery, the views of midwives with bachelors and masters training in midwifery are not captured in this study hence research targeting that group of midwives is necessary to elicit their perceptions about using care plans in the provision of postpartum care.

## Conclusions

Midwives are aware and use the concept of individualized care planning in the provision of postpartum care to their clients though these aspects are often hard to trace due to lack of documentation of the care. They also often employ aspects of clinical reasoning while assessing, diagnosing and treating their clients though this is often demonstrated specifically for clients who may be critically ill or require referral. The benefits of using individualized care plans though known by the midwives are often not harnessed due to high patient loads, lack of time and poor documentation culture among the midwives. The midwives desire more training on the concept and recommend harmonization of the documentation forms and an increase in the midwives employed per facility to enhance the use of the concept in provision of postpartum care.

### Supplementary Information

**Additional file 1: Appendix I.** Interview guide for health workers.

## Data Availability

The data generated and used during the current study can be availed by the corresponding author on reasonable request.

## Abbreviations

HC: Health center
HIV: Human Immunodeficiency Virus
ICP: Individualized care planning
ICPs: Individualized care plans
