# Supplementary material for: Midwives’ perspectives about using individualized care plans in the provision of immediate postpartum care in Uganda; an exploratory qualitative study
Source: BMC Nurs. 2023 Sep 22;22:328. doi: 10.1186/s12912-023-01512-5 (PMC10514976; doi:10.1186/s12912-023-01512-5)
Supplement: Supplementary file 1 — Additional file 1: Appendix I. Interview guide for health workers. [file 12912_2023_1512_MOESM1_ESM.pdf]

Appendix I: Interview guide for health workers

Sub study IV: In-depth interview guide for health workers exploring the midwives' perspectives regarding the use of ICPs in providing postpartum care.

You have been asked to participate in this study exploring the midwives' perspectives on the use of individualised care plans (ICPs) in the provision of postpartum care. This guide has 2 sections namely; introductions, and knowledge/ perceptions about individualized care planning. This interview is expected to take about 1- 11/2 hours to complete. Please feel free to share your opinions. There are no right and wrong answers because we will keep the responses anonymous. The findings of this study will only be used for study purposes. They will not affect your job/work in any way.

Introduction:

1. Tell me about yourself (initials, age, marital status, qualifications, years of practice in midwifery).
2. Tell me about your facility's maternal and new born indicators.
3. How many people work in the maternity unit (labour ward and postnatal section).
4. What roles do you play at this facility?

Knowledge and perceptions about ICP use in postpartum care

1. What do you understand ICP to mean? (Have you ever heard about it? Can you use it in your practice? How confident are you in developing care plans and why?)
2. Do you think it would be beneficial for your postpartum clients? How?
3. How do you use ICP in your postpartum care here?
4. (Probe, if not in use) How do you think this can be incorporated in your care here?
5. What strategies do you think would facilitate or hinder its use in postpartum care here?
